# Side Effects and Perceptions Following COVID-19 Vaccination in Jordan: A Randomized, Cross-Sectional Study Implementing Machine Learning for Predicting Severity of Side Effects

**DOI:** 10.3390/vaccines9060556

**Published:** 2021-05-26

**Authors:** Ma’mon M. Hatmal, Mohammad A. I. Al-Hatamleh, Amin N. Olaimat, Malik Hatmal, Dina M. Alhaj-Qasem, Tamadur M. Olaimat, Rohimah Mohamud

**Affiliations:** 1 Department of Medical Laboratory Sciences, Faculty of Applied Medical Sciences, The Hashemite University, Zarqa 13133, Jordan; 2 Department of Immunology, School of Medical Sciences, Universiti Sains Malaysia, Kubang Kerian, Kelantan 16150, Malaysia; alhatamleh@student.usm.my; 3 Department of Clinical Nutrition and Dietetics, Faculty of Applied Medical Sciences, The Hashemite University, Zarqa 13133, Jordan; aminolaimat@hu.edu.jo; 4 Prince Hamza Hospital, Amman 11947, Jordan; hatmal.malik@yahoo.com; 5 Precision Medical Laboratory, Amman 11954, Jordan; dina.dq96@gmail.com; 6 Faculty of Pharmacy, Zarqa University, Zarqa 13132, Jordan; tamadurmhmd@gmail.com

**Keywords:** SARS-CoV-2 vaccine, COVID-19 vaccine, post-vaccination symptoms, vaccine acceptance, vaccine hesitancy, vaccine anxiety, thrombocytopenia, thrombosis, blood clot, machine learning

## Abstract

Background: Since the coronavirus disease 2019 (COVID-19) was declared a pandemic, there was no doubt that vaccination is the ideal protocol to tackle it. Within a year, a few COVID-19 vaccines have been developed and authorized. This unparalleled initiative in developing vaccines created many uncertainties looming around the efficacy and safety of these vaccines. This study aimed to assess the side effects and perceptions following COVID-19 vaccination in Jordan. Methods: A cross-sectional study was conducted by distributing an online survey targeted toward Jordan inhabitants who received any COVID-19 vaccines. Data were statistically analyzed and certain machine learning (ML) tools, including multilayer perceptron (MLP), eXtreme gradient boosting (XGBoost), random forest (RF), and K-star were used to predict the severity of side effects. Results: A total of 2213 participants were involved in the study after receiving Sinopharm, AstraZeneca, Pfizer-BioNTech, and other vaccines (38.2%, 31%, 27.3%, and 3.5%, respectively). Generally, most of the post-vaccination side effects were common and non-life-threatening (e.g., fatigue, chills, dizziness, fever, headache, joint pain, and myalgia). Only 10% of participants suffered from severe side effects; while 39% and 21% of participants had moderate and mild side effects, respectively. Despite the substantial variations between these vaccines in the presence and severity of side effects, the statistical analysis indicated that these vaccines might provide the same protection against COVID-19 infection. Finally, around 52.9% of participants suffered before vaccination from vaccine hesitancy and anxiety; while after vaccination, 95.5% of participants have advised others to get vaccinated, 80% felt more reassured, and 67% believed that COVID-19 vaccines are safe in the long term. Furthermore, based on the type of vaccine, demographic data, and side effects, the RF, XGBoost, and MLP gave both high accuracies (0.80, 0.79, and 0.70, respectively) and Cohen’s kappa values (0.71, 0.70, and 0.56, respectively). Conclusions: The present study confirmed that the authorized COVID-19 vaccines are safe and getting vaccinated makes people more reassured. Most of the post-vaccination side effects are mild to moderate, which are signs that body’s immune system is building protection. ML can also be used to predict the severity of side effects based on the input data; predicted severe cases may require more medical attention or even hospitalization.

## 1. Introduction

The severe acute respiratory syndrome coronavirus-2 (SARS-CoV-2) emerged in China in late December 2019, causing coronavirus disease 2019 (COVID-19). The virus has rapidly spread across the world, resulting in a worldwide pandemic that has lasted through this moment [1]. Although many therapeutic compounds and drugs have been suggested and repurposed in the fight against COVID-19, they remained as supportive treatment options [2,3].

The process of administering a vaccine to allow the immune system to create protection against an infection is known as vaccination. Vaccines aid in the prevention of illness from incurable conditions by stimulating the body’s adaptive immunity [4]. The only way for resuming normal life and rescuing global economies remains the invention of a vaccine. Therefore, health agencies, university researchers, and medical companies have sparked a race to create and produce effective vaccines [5,6]. Vaccines for COVID-19 are being produced at a rate never seen before. With this unparalleled initiative comes post-marketing safety monitoring and vaccine safety problems [7].

As of 19 April 2021, according to the Milken Institute’s COVID-19 treatment and vaccine tracker, there are 252 COVID-19 vaccine candidates around the world under different vaccine platforms and stages of development [8]. A total of 82 vaccines were under the category of protein subunit, 32 were RNA-based, 30 non-replicating viral vector, 23 replicating viral vector, 22 virus-like particle, 20 inactivated virus, 4 live-attenuated virus, 2 replicating bacterial vector, and 16 unknown. However, only four COVID-19 vaccines were authorized in all countries of the world: BNT162 (Pfizer-BioNTech), mRNA 1273 (Moderna), ChAdOx1 (AstraZeneca), and Ad26.COV2-S (Johnson & Johnson). Other vaccines such as Sputnik V (Gamaleya), BBIBP-CorV (Sinopharm), CoronaVac (Sinovac), and COVAXIN (Bharat Biotech) have also finished phase III clinical trials and have currently been authorized for emergency use in many countries [8].

In Jordan, a country with a population of around 10 million, 685,000 COVID-19 cases with 8200 deaths were reported by 17 April 2021 [9]. The current situation in Jordan is considered worse than the first wave, as the country is suffering from the second wave of COVID-19, which is mainly attributed to the faster transmission of the UK coronavirus variant (known as B.1.1.7). Thus, the government announced stricter measures to combat the outbreak [10]. On the other hand, on 23 December 2020 the Ministry of Health in Jordan has launched the national vaccination campaign and invited everyone who lives in Jordan to register for free vaccination against COVID-19. Till this moment, the number of fully vaccinated people in Jordan is ~122,000 (~1.2%), and the total number of doses given is 665,000 (Jordanian Ministry of Health, 2021).

The rapid development of COVID-19 vaccines, due to the urgency of the pandemic, in addition to technological advances and existing vaccine candidates [11], has contributed to the emergence of many rumors. Vaccine rumors are well-persistent during the post-vaccination period. Rumors that COVID-19 vaccines are linked to various post-vaccination adverse effects (such as infertility) continue to circulate and be debated on various social media sites [12]. Jordanians’ ability to participate in a COVID-19 vaccination has been investigated in previous studies. According to the findings, a low percentage of participants (36.1%) expressed interest in participating in vaccine clinical trials [13]. Another study also found that COVID-19 misinformation and conspiracy beliefs have a negative impact on vaccine hesitancy among Jordanians [14]. This may be a major impediment to the pandemic’s good management. Vaccine hesitancy was linked to a dependence on social media as the primary source of knowledge about COVID-19 vaccines [14]. Therefore, since different vaccines are being used in Jordan’s national vaccination campaign, there is a significant need to assess side effects and perceptions after vaccination in order to tackle vaccine hesitancy and rumors.

This study aimed to look at the side effects that people have identified after receiving various COVID-19 vaccines in Jordan, as well as their impressions. To accomplish that, an online survey was developed and the recorded data was statistically analyzed. Furthermore, machine learning (ML) tools were utilized to build predictive models that involve the implementation of statistical means for learning and predicting outputs like the severity of post-vaccination side effects based on other participants’ responses, demographic and medical data.

## 2. Materials and Methods

### 2.1. Study Design and Participants

A randomized, cross-sectional study was performed from 9 to 15 April 2021 among Jordan inhabitants who received COVID-19 vaccines, regardless of their nationalities, ethnicities, occupations, and places of residence. Adults (18 years and older) were asked to take part in a self-administered online survey (created with Google Forms) that was circulated via social media platforms (i.e., Facebook, Instagram, and WhatsApp).

Potential participants were directed to a page that included a thorough description of the study’s purpose before being asked to agree to a mandatory electronic informed consent form that included statements about voluntary participation and anonymity. This study was approved by The Hashemite University’s Institutional Review Board (IRB), approval code: 3/10/2020/2021, and the World Medical Association Declaration of Helsinki guidance was followed.

### 2.2. The Online Survey Tool

The study’s survey tool (Supplementary Material) was developed after an extensive literature search that included Medline/PubMed, Google Scholar, and other databases. As a result, a wide spectrum of potential post-vaccination side effects was identified and covered in this survey. Several questions were also added to record participants’ demographic data and to assess their general health status before getting vaccinated, as well as their pre- and post-vaccination perceptions about COVID-19 vaccines.

The survey tool was written in English, and it was validated by a panel of experts who provided feedback on the different items of the survey, which were then updated based on their suggestions. The survey was translated into Arabic, the official language in Jordan, before testing and distributing. To test the clarity and comprehensibility of survey content, a pilot study was conducted by including 38 respondents who were excluded from the formal evaluation, and further amendments were carried out based on their comments. The Cronbach’s alpha test of internal consistency was used to assess the reliability of the survey tool. In this study, the test suggested that the survey tool was reliable overall and Cronbach’s alpha was equal to 0.76, which is above 0.70 as a general cut-off limit.

The survey tool’s final edition had four sections. The first section included socio-demographic information and the medical records of participants. The second section looked at the COVID-19 post-vaccination side effects among participants. The third section included several concerns regarding their perceptions, interests, and sources of knowledge about various COVID-19 vaccines. The survey’s final section assessed the signs that lead to hospitalization or medical visits.

### 2.3. Sample Size

The representative sample size was derived from the online Raosoft sample size calculator (Raosoft Inc., Seattle, WA, USA). It was calculated based on a 50% response rate, a 99% confidence interval, and a 5% margin of error with a total 550,000 vaccinated population in Jordan at the end of the study period, as of 15 April 2021 (according to the Ministry of Health, Amman, Jordan). The representative sample size required was 663. In the present study, 2237 respondents participated which represents more than 3-fold of that required sample, which means a convenience sample was used as the sampling tool.

### 2.4. Statistical Analysis

Data were analyzed using the Statistical Package for the Social Sciences (SPSS) version 22.0 (SPSS) (IBM Corporation, Armonk, NY, USA), and vassarstats.net online tools were used for performing the chi-square test. For continuous and categorical variables, the means, standard deviations (SD), and percentages were reported, respectively.

### 2.5. Machine Learning

For the aim of predicting the severity of the side effects after receiving different COVID-19 vaccines, several orthogonal ML tools were used: multilayer perceptron (MLP) (Logistic function), eXtreme gradient boosting (XGBoost), random forest (RF), and K-star (K*). These tools were built using version 4.1.3 of KNIME Analytics Platform (KNIME AG, Zurich, Switzerland). Data was used as 5-fold cross-validation. The input features were based on participants’ responses to all survey’s questions, except questions 10, 11, 12, 16, 17, 18, 20, and 21 (Supplementary Material); these omitted questions were about self-beliefs and emotions, and thus they were excluded to prevent bias in the prediction process. The output was the severity of the post-vaccination side effects (i.e., no, mild, moderate, and severe side effects). 

#### 2.5.1. Random Forest (RF)

RF is a multipurpose ML strategy for classification [15,16,17]. RF is based on an ensemble of decision trees (DTs). Each tree predicts a classification independently and “votes” for the related class, and most of the votes decide the overall RF predictions [18]. RF learner node within KNIME Analytics Platform was implemented with the following settings: splitting criterion is the information gain ratio, which normalizes the standard information gain by the split entropy to overcome any unfair preference for nominal splits with many child nodes, while the number of trees is 100. No limitations were imposed on the number of levels or minimum node size. The accuracy was calculated using out-of-bag internal validation.

#### 2.5.2. eXtreme Gradient Boosting (XGBoost)

XGBoost relies on the ensemble of weak DT-type models to create boosted, DT-type models. This system includes a novel tree learning algorithm, a theoretically justified weighted quantile sketch procedure with parallel, and distributed computing [18,19,20]. We implemented the XGBoost learner node within KNIME Analytics Platform with the following settings: tree booster was implemented with depth wise grow policy, boosting rounds = 100, Eta = 0.3, Gamma = 0, maximum depth = 6, minimum child weight = 1, maximum delta step = 0, subsampling rate = 1, column sampling rate by tree = 1, column sampling rate by level = 1, lambda = 1, Alpha = 0, sketch epsilon = 0.03, scaled position weight = 1, maximum number of bins = 256, sample type (uniform), normalize type (tree), and dropout rate = 0.

#### 2.5.3. Multilayer Perceptron (MLP)

It is an implementation of the RProp algorithm for multilayer feed forward networks [21]. MLP has the capacity to learn nonlinear models in real-time. MLP can have one or more nonlinear hidden layers between the input and output layers. For each hidden layer, different numbers of hidden neurons can be assigned. Each hidden neuron gives a weighted linear summation for the values from the previous layer, and the nonlinear activation function is followed. The output values were reported after the output layer transforms the values from the last hidden layer. We implemented MLP learner node within KNIME Analytics Platform with the following optimized parameters: maximum number of iterations = 100, number of hidden layers = 3, and number of hidden neurons per layer = 100.

#### 2.5.4. K-Star (K*)

The K* is an instance-based classifier. The class of a test instance is based upon the class of those training instances similar to, as determined by some similarity function. It differs from other instance-based learners by using an entropy-based distance function [22]. The default settings were used: average column entropy curve is used for missing mode, and manual blend setting is 20%.

#### 2.5.5. ML Model Evaluation

ML models were evaluated by calculating their accuracies (Equation (1)) and Cohen’s kappa (κ) values (Equation (2)) [23] against the testing sets.

(1) $$\mathrm{Accuracy}\;=\frac{\mathrm{TP}+\mathrm{TN}}{2N}$$

where TP is the true positive (correctly classified predictions), TN is true negative (truly classified predictions), and N is the total number of evaluated cases.

(2) $$K=\frac{P_{0}+P_{e}}{1-P_{e}}$$

where P_0_ is the relative observed agreement among raters (i.e., accuracy), and P_e_ is the hypothetical probability of chance agreement. This was carried out by using the observed data to calculate the probabilities of each observer randomly seeing each category. If the raters are in complete agreement then Cohen’s κ = 1. If there is no agreement among the raters other than what would be expected by chance (as given by P_e_), Cohen’s κ = 0. Negative Cohen’s κ value implies the agreement is worse than random [24].

In 5-fold cross-validation, the process was repeated until all training data points were removed from the training list and predicted at least once. The accuracy was calculated based on comparing classification results with actual reported severities. 

## 3. Results

### 3.1. Demographic Data

The responses of 2213 participants (869 males and 1344 females) were included in the analyses, while twenty-four participants provided inconsistent or incomplete responses (i.e., important items such as informed consent, age, vaccine type, and number of doses were missed), and therefore, they have been excluded. Around one-third of the participants were healthcare workers, and 86% of them were completed their university education (diploma, bachelor or postgraduate studies). Participants were from different age categories and places of residence (Table 1).

### 3.2. Pre-Vaccination

**Figure 1:**
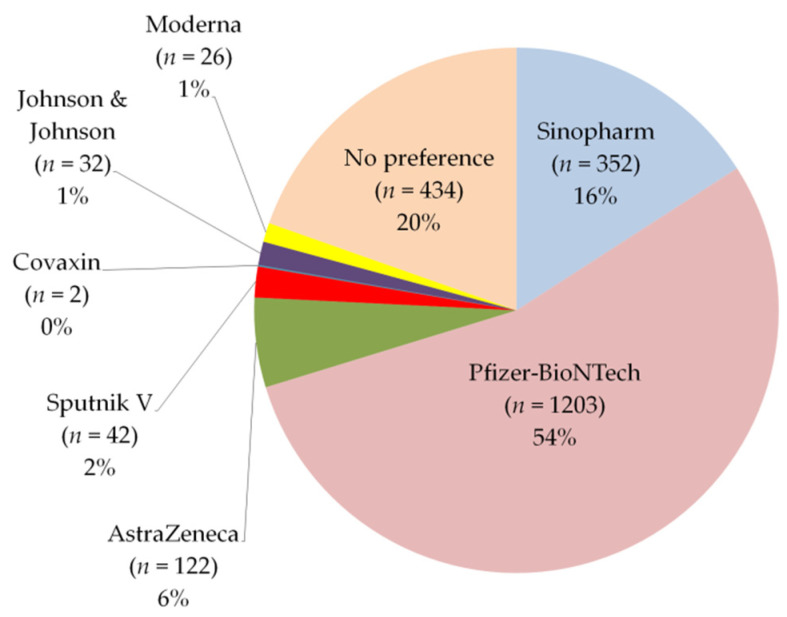
Frequencies of vaccines preferred by participants.

After medical and scientific websites (28%), around 14% of participants showed that Jordan’s public media channels are the second source for COVID-19 vaccine-related information, followed by social media platforms (11%) (Table 2). Based on their personal knowledge and beliefs about COVID-19 vaccines, most of the participants (54%) expressed that they would prefer to get Pfizer-BioNTech vaccine if they can choose before vaccination, 20% had no preferred vaccine, and 16% of them preferred the Sinopharm vaccine (Figure 1).

**Figure 2:**
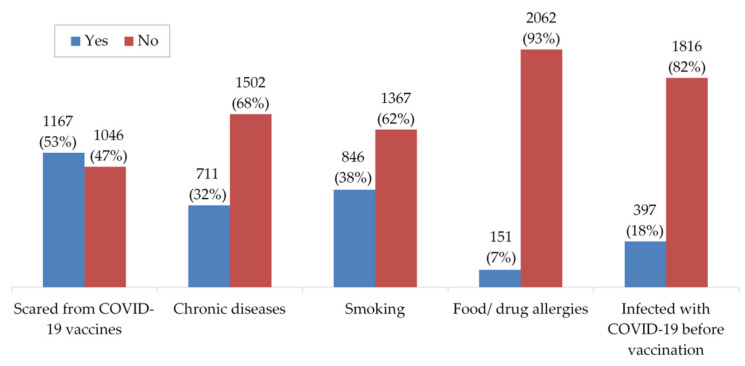
The readiness and health status of participants before getting vaccinated for COVID-19.

Furthermore, more than half of participants reported that they were initially scared from the side effects of COVID-19 vaccines. About 32% of the participants are suffering from chronic diseases, 18% were previously infected with COVID-19, 38% are smokers, and less than 7% have food and drug allergies (Figure 2).

### 3.3. Post-Vaccination

Most of the participants received Sinopharm, AstraZeneca, and Pfizer-BioNTech vaccines, 38.2%, 31%, and 27.34%, respectively, while the rest received other types including Sputnik V, Moderna, Covaxin, and Johnson & Johnson vaccines. However, in terms of full vaccination (i.e., received two doses), participants who received Pfizer-BioNTech vaccine were the majority, compared to their peers who received Sinopharm and AstraZeneca vaccines, respectively (Table 3).

**Figure 3:**
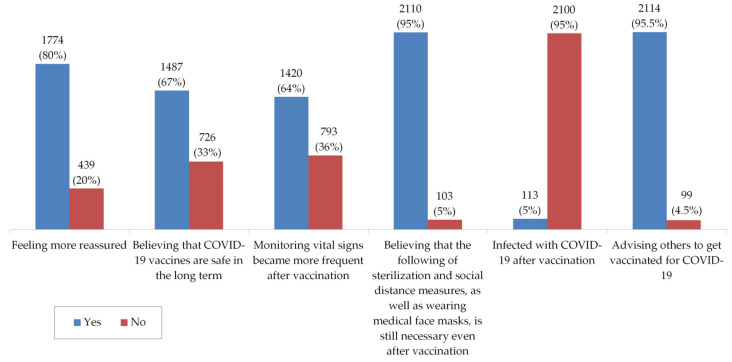
Participants’ perceptions and infection with COVID-19 after vaccination.

Only 5% of the study’s participants reported that they had been infected with COVID-19 after receiving a vaccine. After getting vaccinated, most of the participants felt more reassured (80%) and have advised others to get vaccinated too (95%). Most participants (95%) also believed that keeping prevention measures is still necessary after vaccination, while 67% and 64% of them believed that COVID-19 vaccines are safe for the long term and that they began to monitor their vital signs more frequently after getting vaccinated, respectively (Figure 3). 

**Figure 4:**
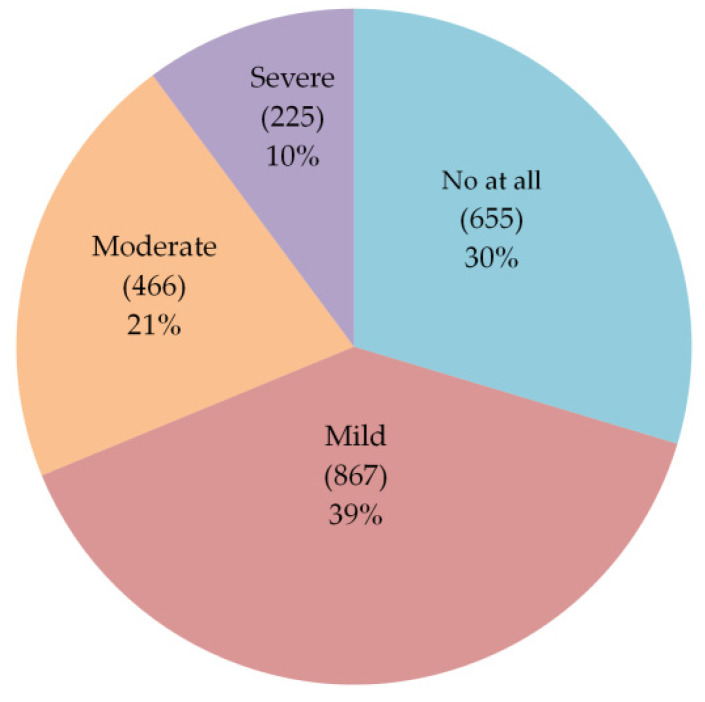
Severity of side effects after vaccination.

**Figure 5:**
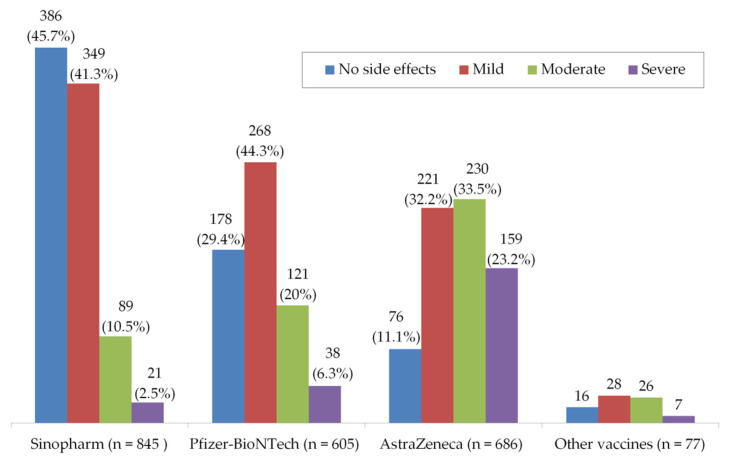
Severity of side effects based on the types of COVID-19 vaccine. Other vaccines are Sputnik V, Moderna, Covaxin, and Johnson & Johnson.

In the present study, out of all participants (2213), 1558 (70%) reported post-vaccination side effects; of them, the majority reported mild (39%) to moderate (21%) side effects. Only 10% suffered from severe side effects (Figure 4). Obviously, the severity of side effects among participants differed based on the type of COVID-19 vaccine. Most of the participants who reported high percentages of moderate to severe side effects (in descending order) were received AstraZeneca, Pfizer-BioNTech, and Sinopharm vaccines, and vice versa for those who recorded no to mild side effects (Figure 5).

On the other hand, despite the types of COVID-19 vaccine, the chi-square (χ^2^) test has shown significant associations between both the presence and number of post-vaccination side effects and the number of received doses (*p* = 0.01 and 0.00, respectively). The presence of side effects was significantly higher at the first dose of COVID-19 vaccines. Furthermore, the number of participants who reported ≥7 different side effects was also higher after the first dose of vaccine. The χ^2^ test also showed that the possibility of infection with COVID-19 after vaccination and the feeling of reassured were both significantly associated with the number of vaccine doses (*p* = 0.00) (Table 4).

#### 3.3.1. Post-Vaccination Side Effects

**Figure 6:**
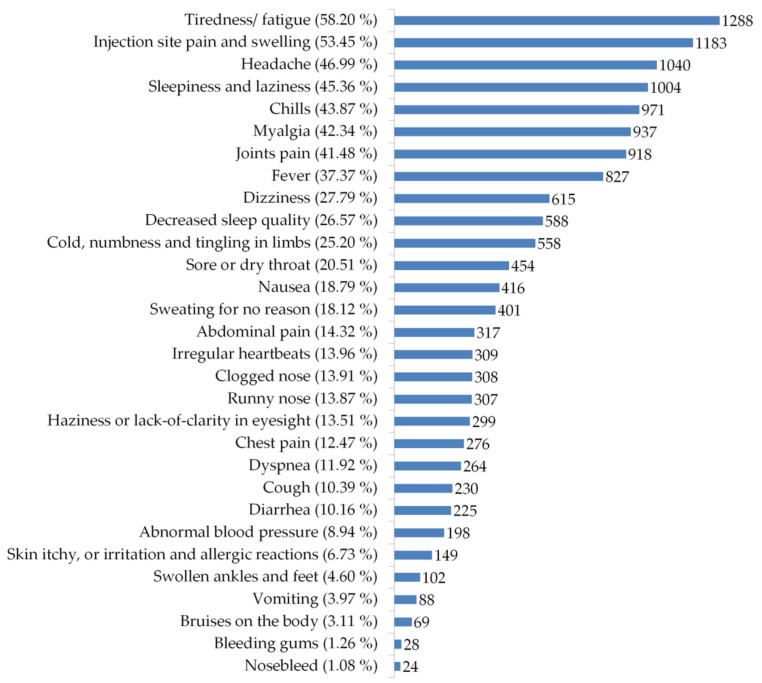
Frequencies of side effects that appeared after receiving COVID-19 vaccines.

A wide spectrum of potential post-vaccination side effects has been assessed among participants in the present study. Figure 6 represents the frequencies of all these side effects, regardless of the type of received COVID-19 vaccine. The most common side effects were fatigue/tiredness (58.20%), injection site pain and swelling (53.45%), headache (46.99%), sleepiness and laziness (45.36%), chills (43.87%), myalgia (42.34%), joints pain (41.48%), and fever (37.37%), respectively. Other side effects were less common among vaccinated participants (Figure 6).

**Figure 7:**
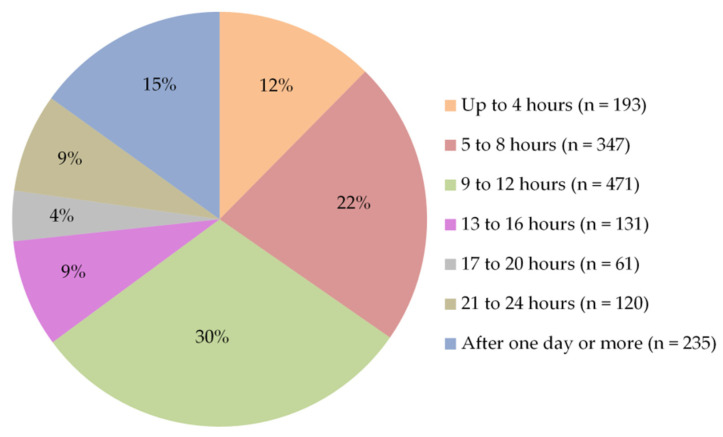
Participants’ answers to the question “How soon did the post-vaccination side effects appear after the injection?”

**Figure 8:**
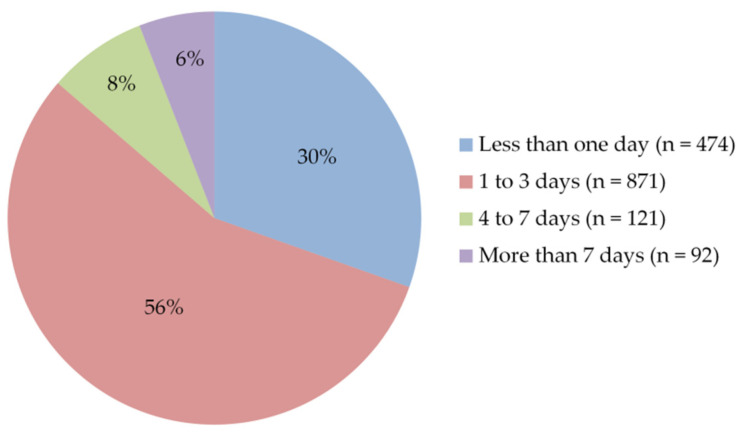
Participants’ answers to the question “How long did the post-vaccination side effects last?”

**Figure 9:**
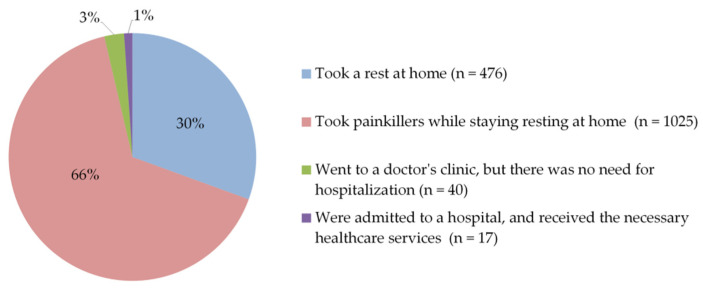
Participants’ acts to relieve post-vaccination side effects.

Regarding the time required for post-vaccination side effects to begin, participants’ responses showed that side effects mostly appeared within 9–12 h of vaccine injection (35%). Around 26% of participants reported that side effects started after 5–8 h, while 15% within up to 4 h (Figure 7). Furthermore, more than half of them (56%) stated that these side effects lasted for one to three days, and 30% for less than one day (Figure 8). To relieve post-vaccination side effects, most of the participants (66%) took painkillers with staying at home, without the need for hospitalization, or even to consult a doctor, while 31% of them took only a rest at home without any medication (Figure 9).

#### 3.3.2. Side Effects and Number of Doses

To address the potential association of post-vaccination side effects with the number of received COVID-19 vaccine doses, the chi-square test (Yate’s corrected) was used (Table 5). The test showed significant associations between the number of doses and the possibility of infection with COVID-19 after vaccination (*p* = 0.00) and a few side effects including nausea (*p* = 0.04), chills (*p* = 0.05), sleepiness and laziness (*p* = 0.00), cold/numbness and tingling in limbs (*p* = 0.00), dizziness (*p* = 0.02), and abnormal blood pressure (*p* = 0.05).

#### 3.3.3. Side Effects and Types of Vaccines

The chi-square test (Yate’s corrected) was also used to assess the potential association of post-vaccination side effects with the different types of COVID-19 vaccine (Table 6). The results showed significant associations between types of vaccine and several side effects including injection site pain and swelling (*p* = 0.00), sleepiness and laziness (*p* = 0.00), chills (*p* = 0.02), clogged or runny nose (*p* = 0.02), and sore or dry throat (*p* = 0.02). The severity and number of side effects were also significantly associated with types of vaccine (*p* = 0.00).

### 3.4. ML to Predict the Severity of Side Effects

For prediction using different ML tools, a confusion matrix was built as indicated in Table 7; Table 8 shows the accuracy, Cohen’s κ, true positive rate (TPR), and true negative rate (TNR) to predict the severity of side effects after receiving different COVID-19 vaccines using different ML tools based on demographic data and other participants responses.

## 4. Discussion

**Figure 10:**
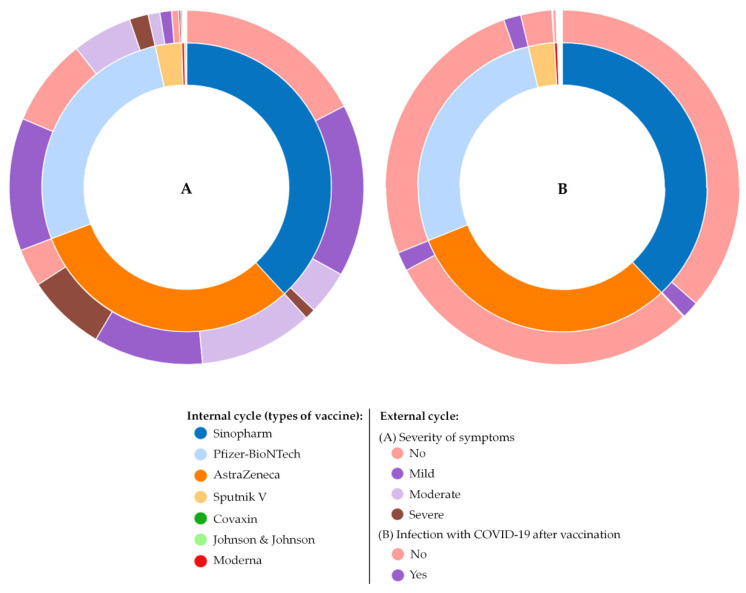
Sunburst illustrations represent the overall findings of the present study: COVID-19 vaccines that participants received, the severity post-vaccination side effects (**A**) and probability of post-vaccination infection (**B**).

In the present study, females (60%) participated more than males. The highest number of people who participated were in the 30–49 years of age category. The most common chronic diseases among the participants were obesity (*n* = 134), joint inflammations (*n* = 79), respiratory diseases (*n* = 67), autoimmune diseases (*n* = 40), thyroid disorders (*n* = 40), dyslipidemia (*n* = 15), cancers (*n* = 12), and osteoporosis (*n* = 3). Different COVID-19 vaccines have been used among participants. As shown in Figure 10A, the main vaccines received by the participants were Sinopharm, Pfizer-BioNTech, and AstraZeneca, respectively. Obviously, the most abundant severe side effects were reported by those who received the AstraZeneca vaccine, while “no symptoms” are mostly reported by those who received Sinopharm vaccine, as shown in Figure 10B.

Not surprisingly, around one-third (*n* = 726, 32.7%) of the participants were healthcare workers. Before vaccine availability, several studies had assessed the acceptance of COVID-19 vaccines among healthcare workers. A study from the United States involved 3479 healthcare workers and showed that only 8% of them were planning to refuse COVID-19 vaccination, suggesting the potential for high uptake [25]. Another study from the Asia-Pacific region showed that around 95% of 1720 healthcare workers were willing to vaccinate against COVID-19 [26]. Therefore, a number of early studies have recruited healthcare workers to assess the potential side effects following COVID-19 vaccination [27,28,29,30].

Similar to findings of studies recently published (Table 9), despite the received COVID-19 vaccine, the most common post-vaccination side effects were fatigue, injection site pain and swelling, headache, sleepiness and laziness, chills, myalgia, joint pain, and fever. However, most of these studies have assessed mainly the post-vaccination side effects of the Pfizer–BioNTech and Moderna vaccines [27,28,29,31,32], while only two studies have focused on the AstraZeneca vaccine [30,33].

There was a significant association between the number of doses of COVID-19 vaccines and the number of the resulting side effects (*p* = 0.00); the side effects tend to be more noticeable after the first dose. However, according to the Centers for Disease Control and Prevention (CDC), side effects after the second dose may be more intense [34]. In fact, due to the urgency of this issue, most of the participants in the previous studies that assessed the post-vaccination side effects had only received the first dose of their COVID-19 vaccines. Therefore, further studies with a greater number of participants who received the second dose are still required in order to confirm this point. Furthermore, the propensity for infection with COVID-19 significantly decreased among participants who received the second dose of the vaccine (*p* = 0.00), implying the value of obtaining the full vaccination. Except for nausea, chills, sleepiness and laziness, cold/numbness and tingling in limbs, dizziness, and abnormal blood pressure, the majority of side effects showed no significant association with the number of doses (Table 5).

Although all COVID-19 vaccines cause similar post-vaccination side effects, both the severity and number of these side effects were significantly associated with vaccine type (*p* = 0.00); AstraZeneca > Pfizer-BioNTech > Sinopharm. The frequencies of some side effects were also significantly associated with vaccine type. The following are the side effects that are significantly associated with types of vaccines (in descending, and with *p*-value): injection site pain and swelling (Pfizer-BioNTech > AstraZeneca > Sinopharm, *p* = 0.00); chills (AstraZeneca > Pfizer-BioNTech > Sinopharm, *p* = 0.02); sore or dry throat, clogged nose, and runny nose (Sinopharm > AstraZeneca > Pfizer-BioNTech, *p* = 0.02); sleepiness and laziness (AstraZeneca > Sinopharm > Pfizer-BioNTech, *p* = 0.00). Overall, these differences may be attributed to the nature of each vaccine and their mechanisms of action.

There are several platforms for viral vaccines development, including live attenuated, inactivated, DNA-based, RNA-based, protein-based, and viral vector-based. Vaccines being developed by each of these strategies have various advantages and disadvantages associated with their efficacy, safety, immunogenicity, and ease of use [35]. In fact, AstraZeneca vaccine consists of ChAdOx1 (replication-deficient simian adenovirus vector) containing the full-length structural SARS-CoV-2 spike (S) protein, with a leader sequence (DNA) fused to a tissue-type plasminogen activator [36]. The S protein plays a crucial role in penetrating host cells and initiating infection of all SARS viruses, and thus it is a major target in the development of vaccines and therapeutics which help in modulating the host response to COVID-19 [1]. The AstraZeneca vaccine expresses a codon-optimized coding sequence for the S protein in the human body, which results in building immunity against SARS-CoV-2 [36]. The Pfizer-BioNTech vaccine is nucleoside-modified mRNAs, formulated in lipid nanoparticles, encoding the S protein of SARS-CoV-2, modified by two proline mutations to lock it in the prefusion conformation [37]. Despite the fact that RNA is a relatively unstable molecule, novel vaccine designs have been developed to improve its stability and protein translation performance, which has resulted in an improved immune response with a high level of neutralizing antibodies [38].

While both AstraZeneca and Pfizer-BioNTech vaccines are developed using next-generation platforms (i.e., DNA- and RNA-based viral vectors), Sinopharm vaccine has been developed using a conventional technology (i.e., inactivated virus). To inactivate virus production, β-propionolactone was thoroughly mixed with the harvested viral solution, while the manufactured vaccine contains total protein with aluminum hydroxide adjuvant [39]. Although DNA vaccines are easy to produce and store with an excellent stability and a limited toxicity [38], the concerns about their safety and immunogenicity still require further investigation. In the rhesus macaque model studies, 5 of 6 lung lobes in the vaccinated group with AstraZeneca vaccine showed a detectable viral load, while after using Sinopharm vaccine there was no detectable viral load [40,41]. However, these studies showed that both AstraZeneca and Sinopharm vaccines conferred effective protection and prevented viral interstitial pneumonia.

In the present study, most of the participants who visited a doctor or have been admitted to a hospital (*n* = 57) suffered from non-serious side effects. However, a total of six vaccinated participants were diagnosed with thrombocytopenia, of whom two were also diagnosed with thrombosis (blood clots) within the first 24 h of getting vaccinated with the second dose of either Pfizer-BioNTech or AstraZeneca vaccine (Table 10). After several reports from different countries across Europe [42], the rare risk of thrombocytopenia and unusual blood clots associated with the AstraZeneca vaccine was confirmed by the European Medicines Agency (EMA) [43]. Recently, concerns started to emerge about the same risks among a few people vaccinated with Pfizer-BioNTech vaccine. A report from Italy shows that a 66-year-old woman has been diagnosed with deep vein thrombosis, despite all blood tests (including platelet count and clotting-related tests) being normal [44]. However, the benefits of these vaccines extremely outweigh their risks, especially knowing the risk of mortality resulting from COVID-19 and that it could cause a greater risk of thrombocytopenia and unusual blood clots [45,46].

Researchers are still searching for possible links between these unusual risks and COVID-19 vaccines. However, previous studies have reported that thrombocytopenia is commonly reported following injection with adenoviral gene transfer vectors [47]. A study showed that the von Willebrand Factor (VWF) and P-selectin are critically involved in a complex platelet–leukocyte–endothelial interplay, resulting in platelet activation and accelerated platelet clearance following adenovirus administration [48]. This probably explains the risk of thrombocytopenia and blood clots associated with adenovirus-based vaccines (i.e., AstraZeneca and Pfizer-BioNTech vaccines). However, it is important to avoid any mistaken identification of other causes as post-vaccination side effects. Therefore, for patients who develop serious symptoms after vaccination, it is necessary to study their health history and full clinical profile to confirm whether these are vaccination-related side effects or not.

Moreover, Pfizer-BioNTech vaccine was the third most commonly received by participants (38.2% Sinopharm, 31% AstraZeneca, 27.34% Pfizer-BioNTech, and 3.46% other vaccines). It was the first vaccine received by participants who are fully vaccinated (61% of 431), and it was the most preferred (54%) vaccine for participants before vaccination. This is probably because it was the first authorized vaccine globally [49], and thus it is one of the most administrated COVID-19 vaccines globally. The reason for this preference could also be attributed to the huge number of online available resources (i.e., studies, web pages, articles, etc.) that recommended its safety and efficacy. Meanwhile, in the present study, the main sources of information about COVID-19 vaccines among participants were medical and scientific websites (Table 2).

Despite the insignificant association between COVID-19 vaccines in the ratio of vaccinated participants who infected with COVID-19 following vaccination (Table 6), it was clear that Sinopharm vaccine has the lowest ratio (33/845), followed by AstraZeneca and Pfizer-BioNTech vaccines (39/686 and 39/605, respectably). However, a recent study by Amit et al. [50] showed that the ratio of vaccinated people infected with COVID-19 after receiving the first dose of Pfizer-BioNTech vaccine to the total number of study’s participants is 22/4081, while in the present study it is 24/342 after the first dose only. This variation in ratios could be due to the fact that Amit et al.’s study [50] involved only healthcare workers; this category of the population is supposed to be well-educated and has a further strict adherence in following prevention measures to avoid COVID-19 infection [51]. In Amit’s study [50], individuals infected with COVID-19 were also tested in the early post-vaccination period (1–10 days), while there was no time limit in the present study.

Despite the fact that the preventive efficacy of COVID-19 vaccines is debated in clinical trials, the knowledge about what happens following vaccination in the real world is still modest, especially among the general population. Thus, knowing what to expect after vaccination will help with public education, dispelling myths, and lowering the apprehension about COVID-19 vaccines [30]. Fear and suspicion, as well as a lack of information about clinical trials, have all been identified as factors that may lead to hesitancy in receiving the COVID-19 vaccines. In addition, the COVID-19 epidemiological situation in the country could also influence vaccine use [13]. In the present study, most of the participants were scared of the COVID-19 vaccine before receiving it (53%). However, the overall picture dramatically changed after vaccination; most of the participants felt more reassured and have advised others to get vaccinated (80%).

Having this in mind, if a country conducts its own independent studies, by its local researchers, on the safety of COVID-19 vaccines, it seems that the public confidence in the vaccines’ safety increased, which accelerates the vaccination process against COVID-19. Therefore, amid the modest participation in Jordan’s national vaccination campaign, the findings of this study could be of interest to the Ministry of Health as they can be utilized during its guided efforts to raise awareness of the safety, effectiveness, and benefits of COVID-19 vaccination. These findings refute the rumors and conspiracy beliefs about the post-vaccination side effects of COVID-19 vaccines, which have a negative impact on vaccine hesitancy among Jordanians [14]. 

On the other side, several ML learners were evaluated, namely, XGBoost, RF, MLP, and K*. Age, gender, and participants’ responses to different questions (Table 8) were used as input descriptors. Clearly from Table 8, learners achieved different accuracies; which prompted us to use Cohen’s κ as additional success criteria of the resulting ML models. Cohen’s κ is a more robust measure than accuracy, as it takes into account the possibility of prediction by chance [52]. Fleiss’s [53] equally arbitrary guidelines characterize Cohen’s κ over 0.75 as excellent, 0.40 to 0.75 as fair to good, and below 0.40 as poor.

RF and XGboost yielded good accuracy and Cohen’s κ values. MLP showed less accuracy and Cohen’s κ values, while in our case K* was the least accurate ML tool and the one with the least Cohen’s κ value. K* can handle noisy data and it requires less time to train the data. However, its performance becomes better with large datasets [54].

Such tools can be implemented in the future for larger databases that include an extensive number of participants and input features, which could efficiently determine people with severe adverse effects who might need hospital admission or medical monitoring. In large databases, feature selection and weighting tools (i.e., genetic algorithms and stepwise forward and reverse methods) can be implemented to select the most relevant predicting subsets of input features.

It is known that some patients with serious life-threatening side effects can conceal their symptoms in order to prevent hospitalization, which may last many days in some cases [55]. Access to these cases may be difficult, particularly during pandemics where a large number of people are infected. ML, on the other hand, is designed to assist in such circumstances by predicting people who may experience serious life-threatening adverse effects based on the type of vaccine, demographic data, and simplified recorded symptoms. These expected cases may necessitate immediate hospitalization and additional medical care. Furthermore, ML may assist in identifying avoidable hospital admissions, reducing the pressure on hospitals.

Moreover, future studies should address the expected background disease events that may be temporarily associated with, but not caused by, COVID-19 vaccination during mass immunization. For example, a study found that the expected incidences of selected coincident background events after the mass immunization against H1N1 influenza in China were as following: 7.71 cases of Guillain-Barré syndrome would be expected to occur within six weeks (from a cohort of 10 million individuals), 126.58 cases of sudden adult death syndrome would be expected to occur within one day of vaccination (from 1 million migrant workers), 1381 cases of preterm labors would be expected to occur within one day after the vaccination (from a cohort of 1 million vaccinated pregnant women with a stillbirth of 196–258 days) [56]. Another study showed that, if a cohort of 10 million people were to be vaccinated against H1N1 influenza in China, 11.5, 80.2, and 481.5 cases of sudden cardiac death would be expected to occur as coincident temporal associations with vaccination, within 1 day, 1 week, and 6 weeks after immunization, respectively [57]. The findings of such studies may help health authorities to assess the safety of COVID-19 vaccines and the effectiveness of mass immunization, and could improve future vaccine education and use.

## 5. Study Implications

The results of the current study may provide an important baseline for increasing the awareness of the general community about COVID-19 vaccines. These findings suggest that health authorities and government should develop and plan appropriate educational strategies to increase the community knowledge and importance of vaccines in eradicating the viral infections which may reduce the vaccine hesitancy due to the lack of knowledge, false religious beliefs, or anti-vaccine misinformation. These results may also increase the public confidence in the safety of COVID-19 vaccines, which accelerate the process of vaccination in Jordan by disproving the rumors and conspiracy beliefs about the post-vaccination side effects of COVID-19 vaccines.

## 6. Study Strengths and Limitations

To the best of our knowledge, this is the first comparative study of Sinopharm, Pfizer-BioNTech, and AstraZeneca vaccines based on the post-vaccination side effects. Since the most common side effects were non-life-threatening, the findings of this study can help in making people more reassured, and thus tackling vaccine hesitancy and conspiracy beliefs. On the other hand, the successful incorporation of ML tools to predict the severity of post-vaccination side effects is a promising approach that can be utilized to predict more about patients’ health based on large medical databases and move towards better healthcare services. This study involved a relatively large sample size and it was 3-fold of the calculated sample size that is required to conflict internal valid study and representative results. However, this study has some limitations such as the use of a self-reported online survey, which may present information bias as a result of side effects bias exposure or misclassification. In addition, the diversity of the sample (based on place of residence) in the current study might be reported due to participation of people who have an internet connection, and the sample was not equally distributed across gender or profession which requires caution in generalizing the findings of this study. In the current study, the side effects of three types of vaccines were compared in the general population; however, other types of vaccines were not yet used in Jordan. Therefore, further study could be required for investigation of side effects of all authorized vaccines. Participants who received each of the three most common vaccines in Jordan may differ by their age since the first used vaccine was Sinopharm vaccine which was given with high priority to elderly and clinically vulnerable people. Since there is a shortage in vaccines, a low percentage of participants were fully vaccinated, particularly those who received AstraZeneca vaccine where its second dose is administered at 12 weeks following the first dose; therefore, only a few participants had received the two doses of AstraZeneca vaccine at the conducting time of this study.

## 7. Conclusions

Despite significant differences in the existence and severity of side effects between these vaccinations, the current study concluded that the approved COVID-19 vaccines are safe, and that getting vaccinated helps people feel more secure. The majority of post-vaccination side effects are mild to moderate, indicating that the body’s immune system is strengthening its defenses. Furthermore, machine learning tools were used to predict the severity of side effects, and it showed that RF, XGBoost, and MLP provided high prediction accuracies of the severity of side effects based on the input data. Predicted extreme cases may require additional medical treatment or even hospitalization.

## Figures and Tables

**Table 1:** Classification of participants involved in the study based on their demographic data.

Variable (*n*, %)	Participants (*n* = 2213)	
	Male *n* = 869 (39.2 %)	Female *n* = 1344 (60.8 %)
Healthcare workers (726, 32.7)	269 (30.91)	457 (69.09)
Age categories (year):		
Less than 20 (8, 0.36)	3 (0.34)	5 (0.37)
29–20 (564, 25.42)	185 (21.26)	379 (28.11)
39–30 (577, 26.01)	254 (29.19)	323 (23.96)
49–40 (490, 22.09)	169 (19.42)	321 (23.81)
59–50 (365, 16.45)	152 (17.47)	213 (15.80)
60 or more (214, 9.64)	107 (12.29)	107 (7.93)
Educational levels:		
High school or less (319, 14.38)	125 (14.36)	194 (14.39)
Diploma/Bachelor’s degree (1453, 65.50)	562 (64.59)	891 (66.09)
Postgraduate studies (446, 20.10)	183 (21.03)	263 (19.51)
Places of residence:		
City (1923, 86.69)	717 (82.41)	1206 (89.46)
Village (266, 11.99)	136 (15.63)	130 (9.64)
Badia (7, 0.31)	4 (0.45)	3 (0.22)
Refugee camp (22, 0.99)	13 (1.49)	9 (0.66)

Badia is a semi-desert region.

**Table 2:** The major sources for information about COVID-19 vaccines among participants.

Sources of Information about COVID-19 Vaccines	*n*	%
Source (1): Medical and scientific websites	632	28
Source (2): Public media	290	13
Source (3): Social media platforms	248	11
Source (4): Colleagues, friends and relatives	96	4
Sources 1, 2, 3, and 4	163	7
Sources 1, 2, and 3	166	7
Sources 2, 3, and 4	75	3
Sources 1, 2, and 4	24	1
Sources 1, 3, and 4	30	1
Sources 2 and 3	94	4
Sources 1 and 2	155	7
Sources 1 and 4	30	1
Sources 3 and 4	48	2
Sources 2 and 4	31	1
Sources 1 and 3	96	4
No information	42	2

**Table 3:** Classification of participants based on types of COVID-19 vaccine.

Vaccine	Participants *n* (%)	First Dose *n* (%)	Second Dose *n* (%)
Sinopharm	845 (38.2)	700 (82.84)	145 (17.16)
AstraZeneca	686 (31%)	669 (97.52)	17 (2.48)
Pfizer-BioNTech	605 (27.34)	342 (56.53)	263 (43.47)
Sputnik V	65 (2.93)	63 (96.92)	2 (3.08)
Moderna	7 (0.31)	5 (71.4)	2 (28.6)
Covaxin	3 (0.13)	1 (33.3)	2 (66.7)
Johnson & Johnson	2 (0.09)	2 (100)	0 (0.00)
Total	2213 (100)	1782 (80.6)	431 (19.4)

**Table 4:** Association of the side effects with the possibility of COVID-19 infection after receiving both doses.

	One Dose (*n* = 1782) *n* (%)	Two Doses (*n* = 431) *n* (%)	*p*-Value
Side effects			0.01 *
Presence	1279 (71.8)	279 (64.7)	
Absence	503 (28.2)	152 (35.3)	
Number of side effects			0.00 *
0	504 (28.3)	151 (35)	
1–6	449 (25.2)	122 (28.3)	
7–12	491 (27.5)	97 (22.5)	
>12	338 (19.0)	61 (14.2)	
Infected with COVID-19 after vaccination	79 (4.4)	34 (7.9)	0.00 *
Feeling more reassured after vaccination	1397 (78.4)	376 (87.2)	0.00 *

* Statistically significant (*p*-value < 0.05).

**Table 5:** Association of each post-vaccination side effect with the number of doses.

		One Dose (*n* = 1782)	Two Doses (*n* = 431)	χ^2^	*p*-Value
The severity of side effects	Non	504	151	2.92	0.09
	Mild	705	162		
	Moderate	378	88		
	Severe	195	30		
Infected after vaccination	Yes	78	35	8.02	0.00 **
	No	1704	396		
Tiredness	Present	1068	220	3.24	0.07
	Absent	210	60		
Fever	Present	688	139	1.32	0.25
	Absent	590	141		
Headache	Present	868	172	3.78	0.05
	Absent	410	108		
Haziness or lack-of-clarity in eyesight	Present	254	45	1.84	0.17
	Absent	1024	235		
Injection site pain and swelling	Present	965	218	0.73	0.39
	Absent	313	62		
Joint pain	Present	766	152	2.6	0.11
	Absent	512	128		
Swollen ankles and feet	Present	76	26	3.72	0.05
	Absent	1202	254		
Myalgia	Present	779	158	1.61	0.20
	Absent	499	122		
Nausea	Present	356	60	4.4	0.04 **
	Absent	922	220		
Abdominal pain	Present	272	45	3.44	0.06
	Absent	1006	235		
Diarrhea	Present	189	36	0.51	0.48
	Absent	1089	244		
Vomiting	Present	74	14	0.13	0.72
	Absent	1204	266		
Bruises on the body	Present	58	11	0.08	0.78
	Absent	1220	269		
Bleeding gums	Present	23	5	0.06	0.80
	Absent	1255	275		
Nosebleed	Present	21	3	0.18	0.67 *
	Absent	1257	277		
Chills	Present	812	159	3.91	0.05 **
	Absent	466	121		
Itchy skin, or irritation and allergic reactions	Present	120	29	0.16	0.69
	Absent	1158	251		
Sweating for no reason	Present	339	62	2	0.16
	Absent	939	218		
Cold, numbness, and tingling in limbs	Present	486	72	14.35	0.00 **
	Absent	792	208		
Dizziness	Present	522	93	5.1	0.02 **
	Absent	756	187		
Clogged nose	Present	249	59	0.3	0.58
	Absent	1029	221		
Runny nose	Present	259	48	1.17	0.28
	Absent	1019	232		
Dyspnea	Present	218	46	0.02	0.89
	Absent	1060	234		
Chest pain	Present	232	44	0.74	0.39
	Absent	1046	236		
Sleepiness and laziness	Present	848	156	10.45	0.00 **
	Absent	430	124		
Irregular heartbeats	Present	255	54	0.02	0.89
	Absent	1023	226		
Abnormal blood pressure	Present	152	46	3.95	0.05 **
	Absent	1126	234		
Sore or dry throat	Present	380	74	0.99	0.32
	Absent	898	206		
Cough	Present	194	36	0.77	0.38
	Absent	1084	244		

* One of the expected cell frequencies is smaller than 5. ** Significant difference.

**Table 6:** Association of each symptom with the types of COVID-19 vaccine.

		Vaccines				χ^2^	*p*-Value
		Sino.	Pfizer.	Astra.	O.		
Severity of side effects	Non	386	178	76	16	12.24	0.00 **
	Mild	349	268	221	28		
	Moderate	89	121	230	26		
	Severe	21	38	159	7		
Infected after vaccination	Yes	33	39	39	2	2.57	0.11
	No	812	566	647	75		
Tiredness	Present	354	319	563	52	0.36	0.55
	Absent	105	108	47	9		
Fever	Present	168	187	434	38	2.33	0.13
	Absent	291	240	176	23		
Headache	Present	276	260	460	44	0.01	0.92
	Absent	183	167	150	17		
Haziness or lack-of-clarity in eyesight	Present	84	57	147	11	1.68	0.19
	Absent	375	370	463	50		
Injection site pain and swelling	Present	281	373	484	45	45.68	0.00 **
	Absent	178	54	126	16		
Joint pain	Present	220	201	456	41	0.01	0.92
	Absent	239	226	154	20		
Swollen ankles and feet	Present	26	19	53	4	0.14	0.71
	Absent	433	408	557	57		
Myalgia	Present	221	219	455	42	0.40	0.53
	Absent	238	208	155	19		
Nausea	Present	107	96	193	20	0.01	0.92
	Absent	352	331	417	41		
Abdominal pain	Present	97	68	141	11	1.80	0.18
	Absent	362	359	469	50		
Diarrhea	Present	55	52	110	8	0.00	1.00
	Absent	404	375	500	53		
Vomiting	Present	17	16	52	3	0.03	0.86
	Absent	442	411	558	58		
Bruises on the body	Present	20	12	34	3	0.39	0.53
	Absent	439	415	576	58		
Bleeding gums	Present	11	1	15	1	2.22	0.14 *
	Absent	448	426	595	60		
Nosebleed	Present	9	2	11	2	0.99	0.32 *
	Absent	450	425	599	59		
Chills	Present	207	238	481	45	5.63	0.02 **
	Absent	252	189	129	16		
Itchy skin, or irritation and allergic reactions	Present	47	31	62	9	0.97	0.32
	Absent	412	396	548	52		
Sweating for no reason	Present	93	71	224	13	0.69	0.41
	Absent	366	356	386	48		
Cold, numbness, and tingling in limbs	Present	140	115	278	25	0.55	0.46
	Absent	319	312	332	36		
Dizziness	Present	168	131	288	28	1.61	0.20
	Absent	291	296	322	33		
Clogged nose	Present	115	70	108	15	5.42	0.02 **
	Absent	344	357	502	46		
Runny nose	Present	111	66	114	16	5.52	0.02 **
	Absent	348	361	496	45		
Dyspnea	Present	71	54	127	12	0.53	0.47
	Absent	388	373	483	49		
Chest pain	Present	60	63	139	14	0.14	0.71
	Absent	399	364	471	47		
Sleepiness and laziness	Present	308	230	420	46	9.06	0.00 **
	Absent	151	197	190	15		
Irregular heartbeats	Present	66	72	158	13	0.34	0.56
	Absent	393	355	452	48		
Abnormal blood pressure	Present	46	50	95	7	0.19	0.66
	Absent	413	377	515	54		
Sore or dry throat	Present	153	100	180	21	5.52	0.02 **
	Absent	306	327	430	40		
Cough	Present	61	57	100	12	0.01	0.92
	Absent	398	370	510	49		
Number of side effects	0	386	178	76	16	18.85	0.00 **
	1–6	205	202	146	17		
	7–12	169	147	248	24		
	>12	86	78	216	19		

Sino., Sinopharm; Pfizer., Pfizer-BioNTech; Astra., AstraZeneca; O., other vaccines including Sputnik V, Moderna, Covaxin, and Johnson & Johnson vaccines. * One of the expected cell frequencies is smaller than 5. ** Significant difference.

**Table 7:** Confusion matrix of the predicted side effects with different severity levels versus the actual numbers.

Predicted Side Effects	Side Effects			
	(A) No Side Effects	(B) Mild	(C) Moderate	(D) Severe
(A) No side effects	TP_A_	E_BA_	E_CA_	E_DA_
(B) Mild	E_AB_	TP_B_	E_CB_	E_DB_
(C) Moderate	E_AC_	E_BC_	TP_C_	E_DC_
(D) Severe	E_AD_	E_BD_	E_CD_	TP_D_

TP, true positive; E, error.

**Table 8:** The accuracy of predicting the severity of side effects after receiving different COVID-19 vaccines using different ML tools based on the demographic data and other participant responses.

	MLP	XGBoost	RF	K*
Accuracy	0.70	0.79	0.80	0.44
Cohen’s κ	0.56	0.70	0.71	0.19
TPR_A_	0.74	1.00	1.00	0.32
TNR_A_	0.92	1.00	1.00	0.72
TPR_B_	0.76	0.79	0.80	0.55
TNR_B_	0.49	0.44	0.44	0.60
TPR_C_	0.52	0.53	0.56	0.42
TNR_C_	0.73	0.70	0.70	0.81
TPR_D_	0.61	0.65	0.66	0.50
TNR_D_	0.86	0.86	0.86	0.88

TPR, true positive ration; TNR, true negative ration. The evaluation metrics were calculated for testing set.

**Table 9:** List of studies that assessed the side effects that occurred following COVID-19 vaccination.

Study ID [Reference]	Country	Study Population	Sample Size	Vaccine Type (*n*)
El-Shitany et al., 2021 [31]	Saudi Arabia	General inhabitants	455	Pfizer-BioNTech
Riad et al., 2021 [27]	Czech Republic	Healthcare workers	877	Pfizer–BioNTech
Kadali et al., 2021a [28]	United States	Healthcare workers	1116	Moderna
Kadali et al., 2021b [29]	United States	Healthcare workers	1245	Pfizer–BioNTech
Jayadevan et al., 2021 [30]	India	Healthcare workers	5396	Covishield (5128), Covaxin (180), Pfizer–BioNTech (44), and Sinopharm (44)
Menni et al., 2021 [33]	United Kingdom	General inhabitants	627,383	Pfizer-BioNTech (282,103) and AstraZeneca (345,280)
Chapin-Bardales et al., 2021 [32]	United States	General inhabitants	3,643,918	Pfizer-BioNTech (1,659,724) and Moderna (1,984,194)

*n*, number of individuals who received the vaccine; Covishield, AstraZeneca vaccine manufactured by Serum Institute, India.

**Table 10:** Data of participants who were diagnosed with thrombocytopenia.

Participant	Gender	Age Category	Vaccine	Number of Doses	Diagnosed with Blood Clots
1	Male	20–29	Pfizer-BioNTech	2	Yes
2	Male	50–59	Pfizer-BioNTech	1	No
3	Male	20–29	AstraZeneca	2	Yes
4	Female	50–59	Sinopharm	1	No
5	Female	>60	Sinopharm	1	No
6	Female	30–39	AstraZeneca	2	No

## Data Availability

All data generated are contained in the present manuscript.

## Supplementary Materials

The following are available online at https://www.mdpi.com/article/10.3390/vaccines9060556/s1, Survey of Side Effects and Opinions Following COVID-19 Vaccination in Jordan.

## References

[1] Hatmal M.M., Alshaer W., Al-Hatamleh M.A.I., Hatmal M., Smadi O., Taha M.O., Oweida A.J., Boer J.C., Mohamud R., Plebanski M. (2020). Comprehensive Structural and Molecular Comparison of Spike Proteins of SARS-CoV-2, SARS-CoV and MERS-CoV, and Their Interactions with ACE2. Cells.

[2] Al-Hatamleh M.A.I., Hatmal M.M., Alshaer W., Rahman E.N.S.E.A., Mohd-Zahid M.H., Alhaj-Qasem D.M., Yean C.Y., Alias I.Z., Jaafar J., Ferji K. (2021). COVID-19 infection and nanomedicine applications for development of vaccines and therapeutics: An overview and future perspectives based on polymersomes. Eur. J. Pharmacol..

[3] Al-Hatamleh M.A.I., Hatmal M.M., Sattar K., Ahmad S., Mustafa M.Z., Bittencourt M.D.C., Mohamud R. (2020). Antiviral and Immunomodulatory Effects of Phytochemicals from Honey against COVID-19: Potential Mechanisms of Action and Future Directions. Molecules.

[4] Swetha G., Rani S.L., Brundha M. (2020). Awareness of the side effects of vaccination among general public. Drug Invention Today.

[5] Haidere M.F., Ratan Z.A., Nowroz S., Zaman S.B., Jung Y.J., Hosseinzadeh H., Cho J.Y. (2021). COVID-19 Vaccine: Critical Questions with Complicated Answers. Biomol. Ther..

[6] Chung Y.H., Beiss V., Fiering S.N., Steinmetz N.F. (2020). COVID-19 Vaccine Frontrunners and Their Nanotechnology Design. ACS Nano.

[7] Petousis-Harris H. (2020). Assessing the Safety of COVID-19 Vaccines: A Primer. Drug Saf..

[8] Milken Institute’s COVID-19 Treatment and Vaccine Tracker. https://covid-19tracker.milkeninstitute.org/#vaccines_intro.

[9] COVID-19 Statistical Report. https://corona.moh.gov.jo/ar.

[10] Jordan Reports Highest Daily Tally of COVID-19 Cases. https://www.reuters.com/article/health-coronavirus-jordan-idUSL1N2LD1JZ.

[11] The Lightning-Fast Quest for COVID Vaccines—And What It Means for Other Diseases. https://www.nature.com/articles/d41586-020-03626-1.

[12] Episode #24—Vaccine Myths vs. Science. https://www.who.int/emergencies/diseases/novel-coronavirus-2019/media-resources/science-in-5/episode-24---vaccine-myths-vs-science.

[13] Abu-Farha R.K., Alzoubi K.H., Khabour O.F. (2020). Public Willingness to Participate in COVID-19 Vaccine Clinical Trials: A Study from Jordan. Patient Prefer Adherence.

[14] Sallam M., Dababseh D., Eid H., Al-Mahzoum K., Al-Haidar A., Taim D., Yaseen A., Ababneh N.A., Bakri F.G., Mahafzah A. (2021). High Rates of COVID-19 Vaccine Hesitancy and Its Association with Conspiracy Beliefs: A Study in Jordan and Kuwait among Other Arab Countries. Vaccines.

[15] Nunez-Lopez Y.O., Retnakaran R., Zinman B., Pratley R.E., Seyhan A.A. (2019). Predicting and understanding the response to short-term intensive insulin therapy in people with early type 2 diabetes. Mol. Metab..

[16] Kappel B.A., De Angelis L., Heiser M., Ballanti M., Stoehr R., Goettsch C., Mavilio M., Artati A., Paoluzi O.A., Adamski J. (2020). Cross-omics analysis revealed gut microbiome-related metabolic pathways underlying atherosclerosis development after antibiotics treatment. Mol. Metab..

[17] Tao C., Pan H., Li Y., Zou Z. (2015). Unsupervised Spectral–Spatial Feature Learning With Stacked Sparse Autoencoder for Hyperspectral Imagery Classification. IEEE Geosci. Remote Sens. Lett..

[18] Rozinajová V., Ezzeddine A.B., Lóderer M., Loebl J., Magyar R., Vrablecová P., Sangaiah A.K., Sheng M., Zhang Z. (2018). Computational Intelligence in Smart Grid Environment. Computational Intelligence for Multimedia Big Data on the Cloud with Engineering Applications.

[19] Ren X., Guo H., Li S., Wang S., Li J., Kraetzer C., Shi Y.Q., Dittmann J., Kim H. (2017). A Novel Image Classification Method with CNN-XGBoost Model. Digital Forensics and Watermarking.

[20] Babajide Mustapha I., Saeed F. (2016). Bioactive Molecule Prediction Using Extreme Gradient Boosting. Molecules.

[21] Riedmiller M., Braun H. A direct adaptive method for faster backpropagation learning: The RPROP algorithm. Proceedings of the IEEE International Conference on Neural Networks.

[22] Wiharto W., Kusnanto H., Herianto H. (2016). Intelligence System for Diagnosis Level of Coronary Heart Disease with K-Star Algorithm. Health Inform. Res..

[23] Hatmal M.M., Abderrahman S.M., Nimer W., Al-Eisawi Z., Al-Ameer H.J., Al-Hatamleh M.A.I., Mohamud R., Alshaer W. (2020). Artificial Neural Networks Model for Predicting Type 2 Diabetes Mellitus Based on VDR Gene FokI Polymorphism, Lipid Profile and Demographic Data. Biology.

[24] McHugh M.L. (2012). Interrater reliability: The kappa statistic. Biochem. Med..

[25] Shekhar R., Sheikh A.B., Upadhyay S., Singh M., Kottewar S., Mir H., Barrett E., Pal S. (2021). COVID-19 Vaccine Acceptance among Health Care Workers in the United States. Vaccines.

[26] Chew N., Cheong C., Kong G., Phua K., Ngiam J.N., Tan B., Wang B., Hao F., Tan W., Han X. (2021). An Asia-Pacific study on healthcare workers’ perceptions of, and willingness to receive, the COVID-19 vaccination. Int. J. Infect. Dis..

[27] Riad A., Pokorná A., Attia S., Klugarová J., Koščík M., Klugar M. (2021). Prevalence of COVID-19 Vaccine Side Effects among Healthcare Workers in the Czech Republic. J. Clin. Med..

[28] Kadali R.A.K., Janagama R., Peruru S., Gajula V., Madathala R.R., Chennaiahgari N., Malayala S.V. (2021). Adverse effects of COVID-19 mRNA-1273 vaccine: A randomized, cross-sectional study on healthcare workers with detailed self-reported symptoms. J. Med. Virol..

[29] Kadali R.A.K., Janagama R., Peruru S., Malayala S.V. (2021). Side effects of BNT162b2 mRNA COVID-19 vaccine: A randomized, cross-sectional study with detailed self-reported symptoms from healthcare workers. Int. J. Infect. Dis..

[30] Jayadevan R., Shenoy R., Anithadevi T. (2021). Survey of symptoms following COVID-19 vaccination in India. medRxiv.

[31] El-Shitany N.A., Harakeh S., Badr-Eldin S.M., Bagher A.M., Eid B., Almukadi H., Alghamdi B.S., Alahmadi A.A., Hassan N.A., Sindi N. (2021). Minor to Moderate Side Effects of Pfizer-BioNTech COVID-19 Vaccine Among Saudi Residents: A Retrospective Cross-Sectional Study. Int. J. Gen. Med..

[32] Chapin-Bardales J., Gee J., Myers T. (2021). Reactogenicity Following Receipt of mRNA-Based COVID-19 Vaccines. JAMA.

[33] Menni C., Klaser K., May A., Polidori L., Capdevila J., Louca P., Sudre C.H., Nguyen L.H., Drew D.A., Merino J. (2021). Vaccine side-effects and SARS-CoV-2 infection after vaccination in users of the COVID Symptom Study app in the UK: A prospective observational study. Lancet Infect. Dis..

[34] Possible Side Effects After Getting a COVID-19 Vaccine. https://www.cdc.gov/coronavirus/2019-ncov/vaccines/expect/after.html.

[35] Kashte S., Gulbake A., El-Amin Iii S.F., Gupta A. (2021). COVID-19 vaccines: Rapid development, implications, challenges and future prospects. Hum. Cell.

[36] Folegatti P.M., Ewer K.J., Aley P.K., Angus B., Becker S., Belij-Rammerstorfer S., Bellamy D., Bibi S., Bittaye M., Clutterbuck E.A. (2020). Safety and immunogenicity of the ChAdOx1 nCoV-19 vaccine against SARS-CoV-2: A preliminary report of a phase 1/2, single-blind, randomised controlled trial. Lancet.

[37] Walsh E.E., Frenck R.W., Falsey A.R., Kitchin N., Absalon J., Gurtman A., Lockhart S., Neuzil K., Mulligan M.J., Bailey R. (2020). Safety and Immunogenicity of Two RNA-Based Covid-19 Vaccine Candidates. N. Engl. J. Med..

[38] Silveira M.M., Moreira G., Mendonca M. (2021). DNA vaccines against COVID-19: Perspectives and challenges. Life Sci..

[39] Xia S., Zhang Y., Wang Y., Wang H., Yang Y., Gao G.F., Tan W., Wu G., Xu M., Lou Z. (2021). Safety and immunogenicity of an inactivated SARS-CoV-2 vaccine, BBIBP-CorV: A randomised, double-blind, placebo-controlled, phase 1/2 trial. Lancet Infect. Dis..

[40] Van Doremalen N., Lambe T., Spencer A., Belij-Rammerstorfer S., Purushotham J.N., Port J.R., Avanzato V.A., Bushmaker T., Flaxman A., Ulaszewska M. (2020). ChAdOx1 nCoV-19 vaccine prevents SARS-CoV-2 pneumonia in rhesus macaques. Nature.

[41] Wang H., Zhang Y., Huang B., Deng W., Quan Y., Wang W., Xu W., Zhao Y., Li N., Zhang J. (2020). Development of an Inactivated Vaccine Candidate, BBIBP-CorV, with Potent Protection against SARS-CoV-2. Cell.

[42] Wise J. (2021). Covid-19: European countries suspend use of Oxford-AstraZeneca vaccine after reports of blood clots. BMJ.

[43] AstraZeneca’s COVID-19 Vaccine: EMA Finds Possible Link to Very Rare Cases of Unusual Blood Clots with Low Blood Platelets. https://www.ema.europa.eu/en/news/astrazenecas-covid-19-vaccine-ema-finds-possible-link-very-rare-cases-unusual-blood-clots-low-blood.

[44] Carli G., Nichele I., Ruggeri M., Barra S., Tosetto A. (2021). Deep vein thrombosis (DVT) occurring shortly after the second dose of mRNA SARS-CoV-2 vaccine. Intern. Emerg. Med..

[45] Bhattacharjee S., Banerjee M. (2020). Immune Thrombocytopenia Secondary to COVID-19: A Systematic Review. SN Compr. Clin. Med..

[46] Wool G.D., Miller J.L. (2021). The Impact of COVID-19 Disease on Platelets and Coagulation. Pathobiology.

[47] Wold W.S., Toth K. (2013). Adenovirus vectors for gene therapy, vaccination and cancer gene therapy. Curr. Gene. Ther..

[48] Othman M., Labelle A., Mazzetti I., Elbatarny H.S., Lillicrap D. (2007). Adenovirus-induced thrombocytopenia: The role of von Willebrand factor and P-selectin in mediating accelerated platelet clearance. Blood.

[49] WHO Issues Its First Emergency Use Validation for a COVID-19 Vaccine and Emphasizes Need for Equitable Global Access. https://www.who.int/news/item/31-12-2020-who-issues-its-first-emergency-use-validation-for-a-covid-19-vaccine-and-emphasizes-need-for-equitable-global-access.

[50] Amit S., Regev-Yochay G., Afek A., Kreiss Y., Leshem E. (2021). Early rate reductions of SARS-CoV-2 infection and COVID-19 in BNT162b2 vaccine recipients. Lancet.

[51] Tripathi R., Alqahtani S.S., Albarraq A.A., Meraya A.M., Tripathi P., Banji D., Alshahrani S., Ahsan W., Alnakhli F.M. (2020). Awareness and Preparedness of COVID-19 Outbreak Among Healthcare Workers and Other Residents of South-West Saudi Arabia: A Cross-Sectional Survey. Front. Public Health.

[52] Landis J.R., Koch G.G. (1977). The measurement of observer agreement for categorical data. Biometrics.

[53] Fleiss J.L., Levin B., Paik M.C. (2003). Statistical Methods for Rates and Proportions.

[54] Mahmood D.Y., Hussein M.A. (2013). Intrusion detection system based on K-star classifier and feature set reduction. IOSR J. Comput. Eng..

[55] Purdey S., Huntley A. (2013). Predicting and preventing avoidable hospital admissions: A review. J. R. Coll. Physicians Edinb..

[56] Wang Y., Wu L., Yu X., Zhao F., Russell A., Song M., Wang W. (2013). The expected number of background disease events during mass immunization in China. PLoS ONE.

[57] Wang Y., Wu L., Yu X., Zhao F., Russell A., Song M., Wang W. (2010). Awareness of the background rate of sudden cardiac death during mass immunization with pandemic H1N1 influenza vaccines increases the intended vaccination rate. Prev. Med..

