# Supplementary material for: Side Effects and Perceptions Following COVID-19 Vaccination in Jordan: A Randomized, Cross-Sectional Study Implementing Machine Learning for Predicting Severity of Side Effects
Source: Vaccines (Basel). 2021 May 26;9(6):556. doi: 10.3390/vaccines9060556 (PMC8229440; doi:10.3390/vaccines9060556)
Supplement: Supplementary file 1 [file vaccines-09-00556-s001.zip › vaccines-1226665-supplementary.pdf]

vaccines MDPI

Survey of Side Effects and Opinions Following COVID-19 Vaccination in Jordan

- This survey aims to study the side effects appeared after receiving COVID-19 vaccines in Jordan.
- This survey aims to study the opinions of people who received COVID-19 vaccines in Jordan.
- This survey targets everyone received the first or second dose of any COVID-19 vaccines in Jordan (regardless of the ethnicity, nationality, age, educational level...etc.).
- Elderly participants can ask a trusted person to assist, or to answer on behalf of them if necessary.
- This survey does not include the names or identifying information about the participants.

SECTION 1: Participant consent

By participating in this study, you are contributing in the awareness-raising efforts about the safety and protection of COVID-19 vaccines, and thus you will help others to protect themselves and to refute any false rumors about these vaccines.

Do you agree to participate in this study?

€ Yes

€ No

SECTION 2: Participant information

1. Gender:

€ Male

€ Female

2. Age category:

€ Less than 20 years

€ 20 - 29 years

€ 30 - 39 years

€ 40 - 49 years

€ 50 - 59 years

€ 60 years or more

3. Educational level:

€ High school or less

€ A Diploma or a Bachelor's degree

€ Postgraduate studies

4. Are you a healthcare worker?

€ Yes

€ No

5. Place of residence:

€ A city

€ A village (rural living)

€ Badia (semi-desert region)

€ A refugee camp

6. Are you suffering from any of chronic diseases?

(*You can select more than one choice*)

€ No

€ Diabetes mellitus

€ Hypertension

€ Cardiovascular diseases

€ Chronic respiratory diseases

€ Obesity

€ Joint inflammations

€ Osteoporosis

€ Autoimmune diseases

€ Thyroid disorders

€ Osteoporosis

€ Cancer

€ Other diseases (please specify): …………………………………………………………

7. Are you a smoker (cigarettes or shisha)?

- € Yes
- € No

8. Are you suffering from an allergy to any types of foods or medicines?

- € Yes
- € No

9. Have you been infected with COVID-19 before vaccination?

- € Yes
- € No

10. Did you feel scared to receive a COVID-19 vaccine before vaccination?

- € Yes
- € No

11. Before vaccination, which type of COVID-19 vaccines did you prefer?

- € AstraZeneca/Oxford
- € Pfizer-BioNTech
- € Sinopharm
- € Johnson & Johnson
- € Moderna
- € Sputnik V
- € Covaxin
- € I have no preference

12. How did you know about COVID-19 vaccines?

- € Government-owned media platforms
- € Social media platforms
- € Friends and relatives
- € Scientific and medical websites
- € I have no information

13. Which type of COVID-19 vaccines have you received?

- € AstraZeneca/Oxford
- € Pfizer-BioNTech
- € Sinopharm
- € Johnson & Johnson
- € Moderna
- € Sputnik V
- € Covaxin

14. How many doses have you received so far?

- € Single dose
- € Two doses

15. Have you got infected with COVID-19 after vaccination?

- € Yes
- € No

16. Do you think that COVID-19 vaccines are safe in the long term?

- € Yes
- € No

17. Do you feel more reassured after vaccination?

- € Yes
- € No

18. Do you think that the following of sterilization and social distance measures, as well as wearing medical face masks, is still necessary after vaccination?

- € Yes
- € No

19. Are you monitoring your vital signs more frequent after vaccination?

- € Yes
- € No

20. Do you advice others to get vaccinated for COVID-19?

- € Yes
- € No

21. Have you noticed any symptoms following vaccination?

- € No symptoms at all (*submit your answers*)
- € Yes, mild symptoms (*complete the next section*)
- € Yes, moderate symptoms (*complete the next section*)
- € Yes, severe symptoms (*complete the next section*)

SECTION 3: Symptoms recorded after vaccination

(*Please note that these symptoms should be appeared suddenly and without known causes*).

22. Have you felt tiredness and fatigue?

- € Yes
- € No

23. Have you experienced decreased sleep quality?

- € Yes
- € No

24. Have you felt a fever?

- € Yes
- € No

25. Have you felt a headache?

- € Yes
- € No

26. Have you experienced haziness or lack-of-clarity in your eyesight?

- € Yes
- € No

27. Have you experienced pain or swelling at the injection site?

- € Yes

€ No

28. Have you felt joints pain?

€ Yes

€ No

29. Have you experienced swollen ankles and feet?

€ Yes

€ No

30. Have you felt muscle pain (myalgia)?

€ Yes

€ No

31. Have you felt nausea?

€ Yes

€ No

32. Have you felt abdominal pain?

€ Yes

€ No

33. Have you experienced diarrhea?

€ Yes

€ No

34. Have you experienced vomiting?

€ Yes

€ No

35. Have you noticed any bruises on your body?

€ Yes

€ No

36. Have you experienced bleeding gums?

€ Yes

€ No

37. Have you experienced a nosebleed?

€ Yes

€ No

38. Have you felt chills?

€ Yes

€ No

39. Have you experienced irritation and allergic skin reactions, or itchy skin?

€ Yes

€ No

40. Have you noticed that your body sweats for no reason?

€ Yes

€ No

41. Have you felt cold, numbness and tingling in limbs?

€ Yes

€ No

42. Have you felt dizzy?

€ Yes

€ No

43. Have you felt a clogged nose?

€ Yes

€ No

44. Have you felt a runny nose?

€ Yes

€ No

45. Have you felt dyspnea?

- € Yes
- € No

46. Have you felt chest pain?

- € Yes
- € No

47. Have you felt over sleepiness or laziness?

- € Yes
- € No

48. Have you felt faster or irregular heartbeats?

- € Yes
- € No

49. Have you experienced an increase or decrease in blood pressure?

- € Yes
- € No

50. Have you felt a sore or dry throat?

- € Yes
- € No

51. Have you experienced a cough?

- € Yes
- € No

52. How soon did the symptoms appear after injection with a COVID-19 vaccine?

- € Up to 4 hours
- € 5 to 8 hours
- € 9 to 12 hours
- € 13 to 16 hours
- € 17 to 20 hours

- € 21 to 24 hours

53. How long did the symptoms last?

- € Less than one day
- € 1 to 3 days
- € 4 to 7 days
- € More than 7 days

54. Please write down any other symptoms you have experienced:

(*optional***)**

…….................................

....

55. How did you act to relieve the symptoms that appeared after vaccination?

- € I took a rest at home (*submit your answers*)
- € I took painkillers while staying rested at home (*submit your answers*)
- € I went to a doctor's clinic, but there was no need for hospitalization (*answer the next section*)
- € I have been admitted to a hospital, and I received the required healthcare services (*answer the next question*)

<u>SECTION 4: After visiting a doctor or hospital</u>

56. Please write down those symptoms caused a visit to doctor or hospitalization:

…….................................

....

57. Have you been diagnosed with any types of thrombosis (blood clots)?

- € Yes
- € No

58. Have you been diagnosed with low platelet count (thrombocytopenia)?

- € Yes
- € No
